# Multi-Response Optimization of Resin Finishing by Using a Taguchi-Based Grey Relational Analysis

**DOI:** 10.3390/ma11030426

**Published:** 2018-03-15

**Authors:** Md. Nahid Pervez, Faizan Shafiq, Zahid Sarwar, Muhammad Munib Jilani, Yingjie Cai

**Affiliations:** 1 Hubei Provincial Engineering Laboratory for Clean Production and High Value Utilization of Bio-Based Textile Materials, Wuhan Textile University, Wuhan 430073, China; nahid.tex92@gmail.com (M.N.P.); faizan22@ymail.com (F.S.); 2 Department of Textile Processing, National Textile University, Faisalabad 37610, Pakistan; zahidsarwar38@yahoo.com (Z.S.); Munibjilani@hotmail.com (M.M.J.)

**Keywords:** resin finishing, multi-response, Taguchi-grey, orthogonal array, ANOVA

## Abstract

In this study, the influence and optimization of the factors of a non-formaldehyde resin finishing process on cotton fabric using a Taguchi-based grey relational analysis were experimentally investigated. An L_27_ orthogonal array was selected for five parameters and three levels by applying Taguchi’s design of experiments. The Taguchi technique was coupled with a grey relational analysis to obtain a grey relational grade for evaluating multiple responses, i.e., crease recovery angle (CRA), tearing strength (TE), and whiteness index (WI). The optimum parameters (values) for resin finishing were the resin concentration (80 g·L^−1^), the polyethylene softener (40 g·L^−1^), the catalyst (25 g·L^−1^), the curing temperature (140 °C), and the curing time (2 min). The goodness-of-fit of the data was validated by an analysis of variance (ANOVA). The optimized sample was characterized by Fourier-transform infrared (FTIR) spectroscopy, thermogravimetric analysis (TGA), and scanning electron microscope (SEM) to better understand the structural details of the resin finishing process. The results showed an improved thermal stability and confirmed the presence of well deposited of resin on the optimized fabric surface.

## 1. Introduction

Cellulosic fibers and more specifically cotton are the most widely used types of fiber due to several advantages, such as the ability to withstand harsh washing solutions, particularly under alkaline conditions, good perspiration absorption characteristics, comfortable wear properties, and the capacity to take up a large variety of dyestuffs [1,2]. However, the inclination to wrinkle under even slight pressing and the lingering maintenance of the wrinkles give cotton clothing a poor wear rating [3]. Ideally, the appearance of garments is imperative to purchasers. To accomplish an attractive appearance, wrinkles may be deliberately created by squeezing operations. On the other hand, wrinkles or short irregular folds that appear unexpectedly on the surface of a fabric or garment during wear are not just unwelcome but also may harm the fabrics because increased wear or breakage may occur along the wrinkles [4,5].

Wrinkle recovery or recuperation is characterized as a fabric property that allows it to recover from the wrinkles. Therefore, wrinkle recovery is one of the essential properties of fabrics influencing the item execution. If the wrinkle remains for a moderately long stretch after it occurred, it affects the fabric’s wearability and quality negatively. The cross-linking of cellulose has for some time been utilized to improve the wrinkle protection of cotton fabrics by diminishing chain slippage under wet conditions [6]. The crosslinkers that are used are also known as resin, easy care, or durable press finishing agents and can be categorized as formaldehyde-based chemicals and formaldehyde-free compounds. The formaldehyde-based chemicals are the most seasoned crosslinkers. Resin finishing was initially conducted using products based on phenol-formaldehyde condensates, methylol melamine, or dimethylol urea. These items resulted in high formaldehyde emissions. Because formaldehyde is a cancer-causing compound, finishing agents with a high formaldehyde emission rate are undesirable. In numerous nations, regulations have been implemented regarding the allowable emissions of formaldehyde. Therefore, there is a need to mitigate the harmful effects of formaldehyde by utilizing a non-formaldehyde resin finishing process. Much effort has been invested to discover non-formaldehyde crosslinking operators for cotton to replace the commonly used *N*-methylol reagents such as zero formaldehyde-based reactants, the commercial products of 1,3-dimethylurea and glyoxal, as well as inorganic phosphates. Polycarboxylic acids such as citric acid are acceptable for crosslinking cotton due to the response with cellulose hydroxyl groups by an anhydride intermediate mechanism [7]. 

There are several parameters influencing the resin finishing process and it is fundamental to upgrade the conditions for a viable resin finishing process. A methodical method for arrangement, execution, and statistical assessment of the procedure is required, and an advanced procedure is required to accomplish the optimum quality attributes of the completed item [8]. Traditional optimization studies that vary one parameter while holding all other parameters fixed are frequently viewed as a thorough but costly approach. The Taguchi design method is an exception to this approach and the use of an analysis of variance (ANOVA) method is a powerful approach that utilizes orthogonal arrays (OAs) to reduce the number of parameters required to decide the optimal settings of the processing parameters. The adequacy of the Taguchi strategy for enhancing quality in the industry has been confirmed. The Taguchi technique facilitates the evaluation of an extensive number of processing parameters while using a fewer number of experimental trials; a single response variable can be tested, while many other test investigate multiple responses [9]. If more than one response has to be evaluated, the multi-attribute decision making (MADM) methodologies are used. The multi-attribute decision making selects the best responses from the current choices by considering multiple responses, which are typically correlated with each other. There are several multi-attribute decision making techniques, such as the technique for the order of preference by similarity to ideal solution (TOPSIS), the grey relational analysis (GRA), the data envelopment analysis (DEA), and the analytic hierarchy process (AHP) [10]. Amongst them, the GRA has been broadly applied in different fields [11].

The GRA was proposed by Deng [12] in 1989 and is a technique for measuring the estimated level among various outcomes utilizing a grey relational grade (GRG). The grey system theory is used to determine vulnerabilities in framework models, examine relationships between frameworks, develop models, and settle on conjectures and choices [13]. The meaning of grey can be defined as the characteristic occurring between the extremes of black and white. In the grey system, black represents the absence of data and white represents complete data in the framework. The purpose of the grey system and its applications is to describe the grey or fuzzy area between the extremes of black and white. The incomplete information is the basic characteristic of a grey system [14]. The GRA is a measurable procedure based on the grey system and transforms multiple qualitative characteristics into single GRGs. By contrasting the computed GRGs, changes in the separate qualitative attributes are determined in accordance with the response evaluations to select an ideal arrangement of the processing parameters [15]. Raza et al. [16] studied multi-response optimization in rhamnolipid production by using the GRA with the Taguchi approach. Rehman et al. [17] also used the Taguchi method for the optimization of enzymatic desizing of cotton fabrics under various chemo-physical conditions. Aslan et al. [18] employed the GRA method to determine the optimal operating parameters of a laboratory scale thickener including feed flow rate, solid percentage, flocculant dosage, and feed well height for the dewatering performance with multiple performance characteristics. Also several resources can be found in literature about multi-response optimization of process parameters by Taguchi-based Grey relational analysis method [19,20,21].

This study is divided into two sections; first, non-formaldehyde resin finishing of cotton fabrics is conducted using an L_27_ OA-based Taguchi design. Second, different processing parameters including three levels of the concentrations of the resin (g·L^−1^), catalyst (g·L^−1^), and the softener (g·L^−1^), as well as the curing temperature (°C) and curing time (min) are investigated to determine the optimum operating parameters of a resin finishing process to achieve the optimum multiple characteristics by using the Taguchi-based GRA; this approach has not been reported previously in the literature.

## 2. Materials and Methods

### 2.1. Materials

A 100% bleached plain weave cotton fabric (ends per inch × picks per inch = 85 × 52; yarn count 40^s^ Ne), with a fabric weight of 102 g·m^−2^ was used in this study. The cross-linkers Knittex RCT^®^ (modified dihydroxyethyleneurea) and Knittex^®^ Catalyst Mo were used as catalysts and both were obtained from Huntsman (The Woodlands, TX, USA). Siligen GL (nonionic polysiloxane by BASF, Basel, Switzerland) was used as a softener.

### 2.2. Design of Experiments

The five controllable factors that are considered in this research with three levels per factor are shown in Table 1. Taguchi’s experimental design (OA L_27_) was constructed by using Minitab^®^ 17 statistical software (Minitab Inc, Coventry, UK) and the details are provided in Table 2. The resin finishing was carried out on a laboratory padder and stenter according to the experimental design. The pH of the solution was maintained at 5.5 by adding a small quantity (3 to 4 drops) of acetic acid. All fabric samples were padded with a wet pick-up of 75%, were dried at 120 °C for 3 min, and then cured according to the experimental design. Then the fabric was removed from the curing chamber, cooled at room temperature.

### 2.3. Measurements

The physical tests on the fabrics were performed at 65% relative humidity and 20 °C using standard test procedures. The crease recovery of the fabric samples was assessed by the American Association of Textile Chemists and Colorists (AATCC) Test Method 128-1974 [22]. For the tearing test, an intensity tearing tester (Elmendorf type) was used according to the ASTM D1424 standard [23]. The whiteness index (WI) was measured according to the ASTM Test Method E 313 [24] using a HUNTER Lab D25 made in the (11491 Sunset Hills Rd, Reston, VA 20190, USA). The mean values of the results were used for the statistical analyses.

### 2.4. Taguchi Approach

Genichi Taguchi [25] developed a method based on an OA of experiments, which results in a lower variance for the experiment to determine the optimum settings of the processing parameters. In order to evaluate the processing parameters, the Taguchi method uses the signal-to-noise (S/N) ratio as a performance criterion or quality index expressed in decibels (dB). The S/N ratio is a logarithmic function of the desired output that serves as an objective function for the optimization. The S/N ratio is the ratio of the mean (signal) to the standard deviation (noise). This ratio is a measure of robustness used to identify the parameters that reduce the variability in a product or process by minimizing the effects of uncontrollable factors. There are three types of S/N ratios, that are the-lower-the-better, the-higher-the–better, and the-nominal-the-better. A higher S/N ratio represents a better product quality. The concept is to maximize the S/N ratio by minimizing the effect of the random noise factors, which have an important impact on the process performance [26]. The S/N ratio with a lower-the-better characteristic, with a higher-the-better characteristic, and with a nominal-the-better characteristic can be expressed as in Equations (1)–(3), respectively.

(1) $$S/N=-10\log\left(\frac{1}{n}\sum_{i=1}^{n}y_{i}^{2}\right)$$

(2) $$S/N=-10\log\left(\frac{1}{n}\sum_{i=1}^{n}\frac{1}{y_{i}^{2}}\right)$$

(3) $$\frac{S}{N}=10\log\left(\frac{{\bar{y}}^{2}}{s^{2}}\right)$$

where *y_i_* is the *i*th experiments in orthogonal array, *n* is the total number of the tests, ${\bar{y}}^{2}$ is the average of data observed and *s*^2^ the variation. 

### 2.5. Crease Recovery Angle

The Taguchi technique for determining the optimal settings of the processing parameters focuses only on a single response. However, in many cases, multiple quality characteristics need to be identified. In addition, the manufacturer has to produce items and maintain a balance between quantity and quality with minimum production costs to earn a maximum profit; therefore, the optimization of multiple quality characteristics is required. The GRA is well suited for this approach since a mathematical technique optimizes two or more quality characteristics. The GRA approach is based on the calculation of the GRGs, which represent the level of correlation between the reference sequence and the comparability sequence of multiple performance characteristics. If two sequences are identical, the value of the GRG is equal to 1. Hence, the higher the GRG value, the better the corresponding multiple performance characteristics are.

### 2.6. Data Pre-Processing

In GRA, the function n of the factors is neglected when the range of the sequence or the standard value is large. However, if the factors, goals, and directions are different, the GRA might produce incorrect results. Therefore, one has to pre-process the data that are related to a group of sequences, which is called the grey relational generation’ [27]. Data pre-processing consists of transferring the original sequence to a comparable sequence. It is required because the ranges and units may differ for different data sequences. This is also necessary when the directions of the target in the sequence are different. For this purpose, the experimental results were normalized to a range between zero and one. Depending on the response of the data sequence, i.e., either the-larger-the-better or the-smaller-the-better, there are various methodologies of GRA data pre-processing that have been suggested by researchers [28]. For the larger-the-better characteristic, the sequence can be normalized as in Equation (4).

(4) $${x_{i}}^{*}\left(k\right)=\frac{y_{i}\left(k\right)-\min y_{i}\left(k\right)}{\max y_{i}\left(k\right)-\min y_{i}\left(k\right)}$$

where x_i_* (k) is the sequence after data pre-processing and *y_i_* (k) is the original sequence of the mean value of the responses; k = 1 for the responses; *i* is for the experiment number. 

### 2.7. Calculating the Grey Relational Coefficient and GRG

After the normalization, the deviation sequence is the next step in the GRA and is computed using Equation (5). The grey relational coefficient is calculated to determine the relationship between the optimal and the actual normalized results and is expressed as in Equation (6) [29].

Δ_0*i*_ (k) = |x_0_* (k) − x_i_* (k)| (5)

(6) $$\xi_{i}\left(k\right)=\frac{\Delta_{\min}+\Psi \Delta_{\max}}{\Delta_{0i}\left(k\right)+\Psi \Delta_{\max}}$$

where $\Delta_{0i}\left(k\right)$ is the deviation sequence of the reference sequence x_0_* (k) and the comparability sequence x_i_* (k) and Ψ is a distinguishing coefficient, 0 ≤ Ψ ≤ 1. The value of Ψ is set to 0.5 to maintain equal weights for all parameters. Based on the grey relational coefficients of each response variable, the GRG γ_i_ is obtained by averaging the grey relational coefficient corresponding to each experiment (Equation (7)). The overall evaluation of the multiple performance characteristics is based on the GRG.

(7) $$\gamma_{i}=\frac{1}{n}\sum_{i=1}^{0}\xi_{i}\left(k\right)$$

where $\gamma_{i}$ is the GRG for the *i*th experiment and n is the number of performance characteristics. The next step is the prediction and verification of the quality characteristics using the optimal level of the design parameters. The predicted GRG is calculated using Equation (8).

(8) $$\gamma_{\mathrm{predicted}}=\gamma_{m}+\sum_{i=1}^{q}\left(\gamma_{i}-\gamma_{m}\right)$$

where $\gamma_{m}$ is the mean GRG, $\gamma_{i}$ is the GRG at the optimal level, and *q* is the number of process parameters that affect the responses.

### 2.8. ANOVA

The ANOVA is a statistical approach to analyze the influence of selected factors on the output response and distributed the variability of the response variables among the available factors. In many types of analyses, it is necessary to identify the parameters that are responsible for a wide variation in the output responses and to quantify the variation. This technique is applied for evaluating the differences between the available factors and is also used to quantify the chosen parameters contribution towards the output [30]. The percent of the contribution of the process parameters to the total sum of the squared deviations was used to evaluate the importance of the parameter changes on the performance characteristics. In addition, an F test was also used to determine which process parameters had a significant effect on the performance characteristics. The change in the process parameter has a significant effect on the performance characteristic when the F-value is large. The inferences derived from the ANOVA table are used to identify which input parameters are responsible for changes in the process performance; by controlling these parameters, the process can be improved. In this technique, more importance is placed on data variance than data analysis [31].

### 2.9. Characterization

In order to confirm the presence of the non-formaldehyde finish on the cotton fabric, Fourier-transform infrared (FTIR) measurements were performed with a Bruker Tensor 27 (Bremen, Germany) spectrometer in normal transmission mode. The thermal stability of the cotton fabrics was analyzed by thermogravimetric analysis (TGA) using a thermogravimetric analyzer (STA-449C; Netsch Instrument Co., Ltd., Selb, Germany). The surface morphological structures of samples were characterized by using scanning electron microscope (Hitachi S-4700 SEM, JEOL Ltd., Tokyo, Japan).

## 3. Results and Discussion

The GRA coupled with the Taguchi method requires the conversion of the experimental data to S/N ratio values. In this study, the crease recovery angle (CRA), tearing strength (TE), and whiteness index (WI) of the resin finished fabric were analyzed to determine the effect of the resin finishing process parameters. The experimental results were transformed into S/N ratios using the Minitab statistical software and the approach is reported step-by-step. According to the Taguchi’s method, “the larger-the-better analysis” was selected for investigating the effects of the factors on the responses, meaning that the higher the value, the better the response is. This section has two sub-sections. The first sub-section describes the results of the Taguchi method experiments and the second sub-section describes the results of the GRA.

### 3.1. Effect of Process Parameters on the CRA

Creases are characterized as the fabric’s distortions due to its viscoelastic properties. More specifically, the ability to withstand the breaking and re-formation of the hydrogen bonds in the amorphous regions of the cotton fibers plays a role in determining the crease formation and crease resistance properties of the fabrics. This kind of deformation not only has undesirable effects on the fabrics’ appearance but also results in lower fabric wear quality [32]. In order to determine the effects of the process parameters on the CRA, experiments were conducted using the L_27_ OA (Table 2). The experimental data and S/N ratios of the crease recovery test of the fabrics are shown in Table 2. The response table for the S/N ratio results of the CRA for the resin finished fabrics is given in Table 3. It helps to analyze the effect of the control factors based on the delta statistics. The delta statistics are defined as the highest average value minus the lowest average value of the individual factors and the delta ranks are assigned based on these values; a higher delta value represents rank 1, the second highest represents rank 2, etc. This analysis helps to obtain more data about the process under investigation and the highest delta value represents the most influential factor on the CRA. The results indicate that the resin concentration has the strongest effect on the CRA with a delta value of 0.57. The curing temperature is the second most important factor with a delta value of 0.17, followed by the softener, the curing time, and the catalyst with delta values of 0.13, 0.12, and 0.03, respectively. 

**Figure 1:**
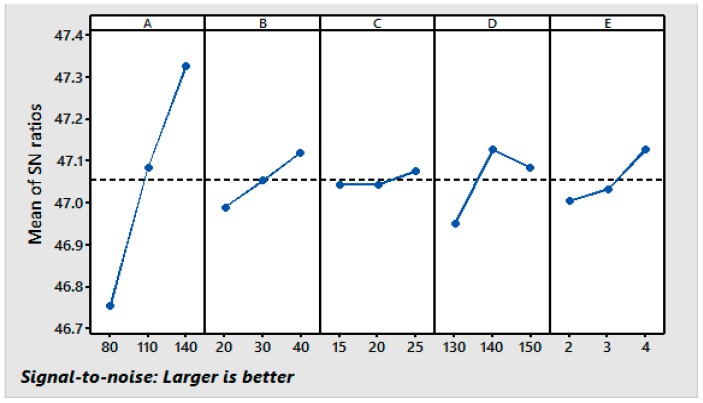
Main effect plots for S/N ratios [Response: CRA].

Using the responses shown in Table 3, graphs of the main effects for the CRA were generated and are shown in Figure 1. It can be observed that the CRA increases with the increase in the concentrations of the resin, the polyethylene softener, the catalyst, and the curing time and decreases with the increase in the curing temperature. The addition of the catalyst and softeners to the finishing bath results in full swelling of the cotton fabric and facilitates the penetration of the finishing agent into the fibers; this increases the cross-linking positions and results in a considerable increase in the CRA. The increase in the CRA is attributed to the increased crosslinking of the cellulose chains under these conditions. The CRA decreases with the increase in the curing temperature from 130 °C to 150 °C, but at 140 °C, the CRA is high because the higher curing temperature may hinder the crosslinking of the cellulose chains, thus reducing the CRA. It can be seen from Figure 1 that the third levels of the resin (A_3_), the softener (B_3_), and the catalyst (C_3_), the second level of the curing temperature (D2), and the third level of the curing time (E_3_) result in the maximum values of the CRA. The S/N ratio analysis suggests that the same levels of the variables (A_3_, B_3_, C_3_, D_2_, and E_3_) are the optimum levels for the maximum CRA in the resin finishing process.

In order to investigate which parameters significantly affect the CRA and to determine the percent contribution of operational variables to the response, an ANOVA was performed (Table 4). Table 4 shows that the resin concentration has the highest influence (81.09%) on the CRA followed by the curing temperature (8.23%), the polyurethane softener (4.21%), the curing time (4.18%), and the catalyst concentration (0.38%). The *p*-values are less than 0.05 for all parameters except for the catalyst concentration (95% confidence level).

### 3.2. Effect of Processing Parameters on the TE

The TE is one of the important aspects of a finished fabric. It refers to the thread-by-thread rupture of a fabric along a line. The measurement of the TE is a widely used parameter in today’s garment industry because it is a measure of the serviceability of the fabric [33]. Due to the importance of this criterion, research on predicting the tear force in the fabric is important to understand this complex phenomenon. However, durable press finishing adversely affects the TE of woven cotton fabrics [34]. As a result, it is worth to investigate the relationship between the durable press performance and the TE of the woven cotton fabrics. In order to determine the effects of the processing parameters on the TE, experiments were conducted using the L_27_ OA (Table 2). The experimental data and the S/N ratios of the TE of the fabrics are shown in Table 2. According to Taguchi’s method, “the larger-the-better analysis” was selected for investigating the effects of the factors on the fabrics’ TE performance; a higher TE is better. The response table for the SN ratio analysis of the TE of the resin finished fabrics is given in Table 5. The results indicate that the resin concentration has the strongest effect on the TE with a delta value of 0.56. The catalyst concentration is the most second important factor with a delta value of 0.10, followed by the curing time, curing temperature, and softener with delta values of 0.08, 0.07, and 0.06, respectively.

**Figure 2:**
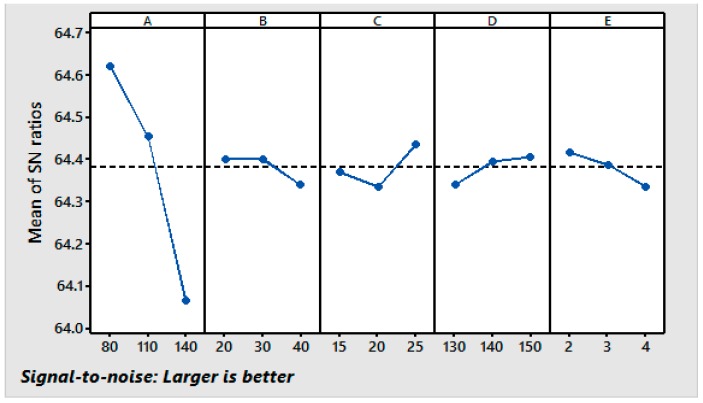
Main effect plots for S/N ratios [Response: TE].

Using the responses shown in Table 5, the graphs of the main effects for the fabric’s TE were generated and are shown in Figure 2. The fabric’s TE decreases with increases in the concentrations of the resin, softener, and catalyst and increases in the curing time and curing temperature. The decrease in the TE may be attributed to increased crosslinking of the cellulose chains, which reduces yarn slippage to resist tearing. Another possible reason for the loss of the fabric strength is the acid-catalyzed fiber degradation at higher curing times. However, the loss of the TE as a result of the crosslinking is minimized by the addition of the catalyst and higher temperatures, which improves the chain slippage. It can be seen from Figure 2 that the first level of resin (A_1_), the second level of softener (B_2_), the third level of the catalyst (C_3_), the third level of the curing temperature (D_3_), and the first level of the curing time (E_1_) provide the maximum value of the TE. The S/N ratio results suggest the same levels of the variables (A_1_, B_2_, C_3_, D_3_, and E_1_) as the optimum levels for the maximum TE in the resin finishing process.

In order to investigate which processing parameters significantly affect the TE and to determine the percent contribution of the operational variables to the response, an ANOVA was performed (Table 6). Table 6 shows that the resin concentration has the highest influence (89.30%) on the TE, followed by the catalyst concentration (2.84%), the curing time (1.82%), the curing temperature (1.38%), and the polyurethane softener concentration (1.35%). The *p*-values are less than 0.05 for all parameters except for the polyurethane softener concentration and the curing temperature (95% confidence level).

### 3.3. Effect of the Processing Parameters on the WI

The fabric’s whiteness is one of the most important indicators of cleaning performance in a home laundry. The WI of the samples is measured to evaluate the influence of a laser treatment on the whiteness. The higher the WI, the greater the whiteness of the measured sample is. Whiteness is an important attribute of colors in the textile industry [35]. In order to determine the effect of the processing parameters on the WI, experiments were conducted using the L_27_ OA (Table 2). According to Taguchi’s method, “the larger-the-better analysis” was selected for investigating the effects of the factors on the fabrics’ WI performance; a higher WI is better. The response table for the S/N ratio analysis of the WI for the resin finished fabrics is given in Table 7. The results indicate that the resin concentration has the strongest effect on the WI with a delta value of 0.34. The curing time is the second most important factor with a delta value of 0.17, followed by the softener, curing temperature, and catalyst concentration with delta values of 0.16, 0.15, and 0.05, respectively.

**Figure 3:**
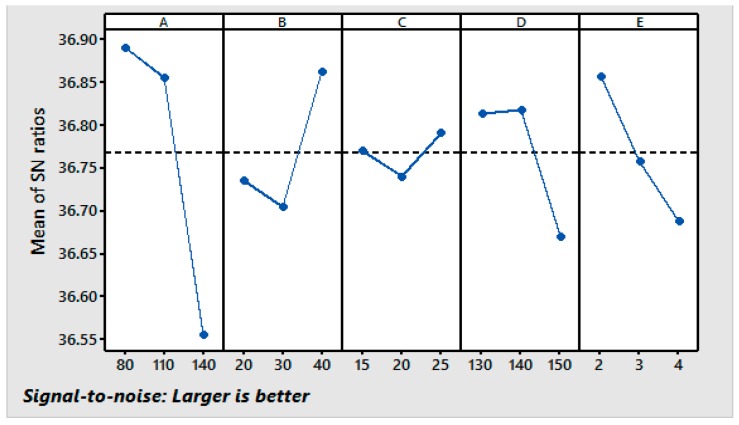
Main effect plots for the S/N ratios [Response: WI].

Using the responses shown in Table 7, graphs of the main effects for the fabric’s WI were generated and are shown in Figure 3. It can be seen that the WI decreases with increases in the resin concentration, the curing temperature, and the curing time and increases with an increase in the concentration of the in polyethylene softener and the catalyst. Higher curing temperatures caused a higher CRA (Figure 1) but curing temperatures higher than 140 °C resulted in a decrease in the WI. At higher curing temperatures, more crosslinking occurs by making use of the energy generated by the vibration of the water molecules and other ionic components, e.g., crosslinking agent and catalyst; therefore, the treated fibers discolor and the WI decreases [36]. It can be seen from Figure 3 that the first level of the resin (A_1_), the third level of the softener (B_3_), the third level of the catalyst (C_3_), the second level of the curing temperature (D_2_), and first level of the curing time (E_1_) provide the maximum value of the WI. The S/N results suggest the same levels of the variables (A_1_, B_3_, C_3_, D_2_, and E_1_) as the optimum levels for the maximum WI in the resin finishing process.

Similar to the analysis of the other parameters, an ANOVA was performed. Table 8 indicates that the resin concentration has the highest influence (51.42%) on the WI, followed by the curing time (11.0%), the curing temperature (10.70%), the polyurethane softener (10.60%), and the catalyst concentration (1.01%). The *p*-values are less than 0.05 for all parameters except for the catalyst concentration (95% confidence level).

### 3.4. Multi-Objective Characteristic Optimization by GRA

**Figure 4:**
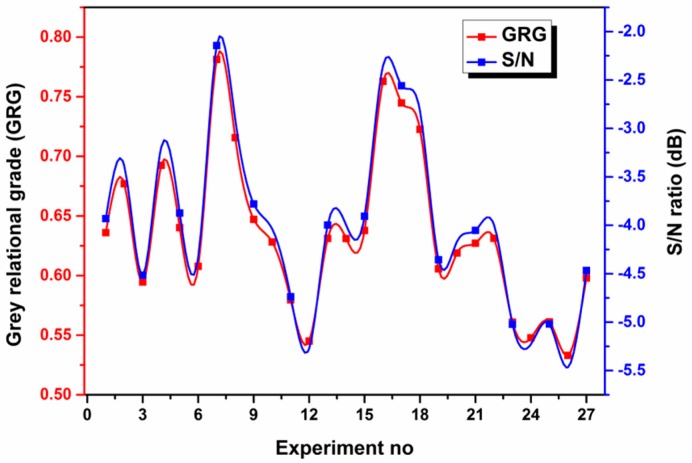
GRG and its S/N ratio.

A GRA is used for incomplete real-world problems when there are no black and white solutions and grey areas are used to describe the outcomes. The GRA has been proved to be successful in optimizing the responses of many materials [37,38]. In the GRA, the generation of the grey relationships was applied to the experimental data related to the quality characteristics; the results were used to obtain the GRG to rank the data series. This subsection explains the results of this method. Table 9 shows the normalized results for all responses after data pre-processing using Equation (4). The response values are in the range of 0 to 1. After the normalization, the deviation sequence is the next step in the GRA and is computed using Equation (5). Table 10 shows the absolute difference of the array Δ_0*i*_ (k) and the grey relational coefficient $\xi_{i}\left(k\right)$. The average value of the grey relational coefficient $\xi_{i}\left(k\right)$ is the GRG $\gamma_{i}$ for the performance characteristics. The grey relational coefficients are calculated using Equation (6). Table 11 shows the GRGs based on Equation (7) for each experiment using the L_27_ OA. A higher GRG indicates that the result is closer to the ideal normalized value. Table 11 also shows the S/N ratios of the GRGs and Figure 4 shows further details for these parameters.

The response table for the GRG (Table 12) lists the priority of the parameters that influence the process. The results show that the resin concentration has the strongest effect on the CRA with a delta value of 0.6658. The softener is the second most important factor with a delta value of 0.6741, followed by the curing temperature, the catalyst, and the curing time with delta values of 0.6692, 0.6551, and 0.6589, respectively. The response table is also helpful for determining the optimum conditions of the input parameters. For each input parameter, the corresponding level value for the highest GRG was noted as the optimum point [20].

**Figure 5:**
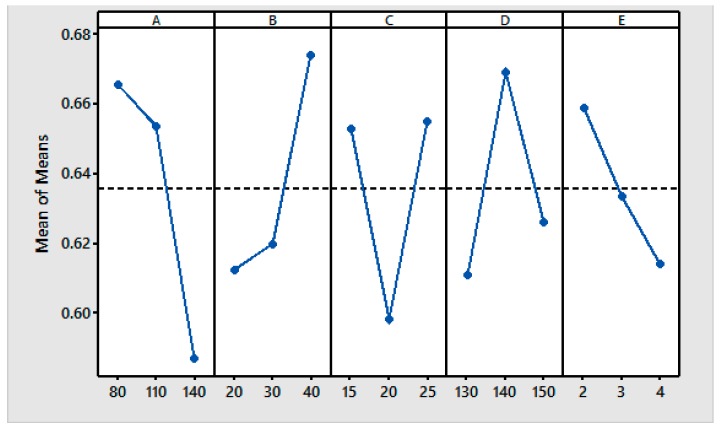
Main effect plots for the means [Response: GRG].

The optimum conditions of the input parameters were further analyzed using the main effects plots for the GRG (Figure 5). A higher GRG value indicates a greater influence of the particular parameter at that level. The maximum value in each graph specifies the optimum level of that particular parameter. It can be seen from Figure 5 that the first level of resin (A_1_), the third level of the softener (B_3_), the third level of the catalyst (C_3_), the second level of the curing temperature (D_2_), and the first level of the curing time (E_1_) provide the maximum values of the GRG.

**Figure 6:**
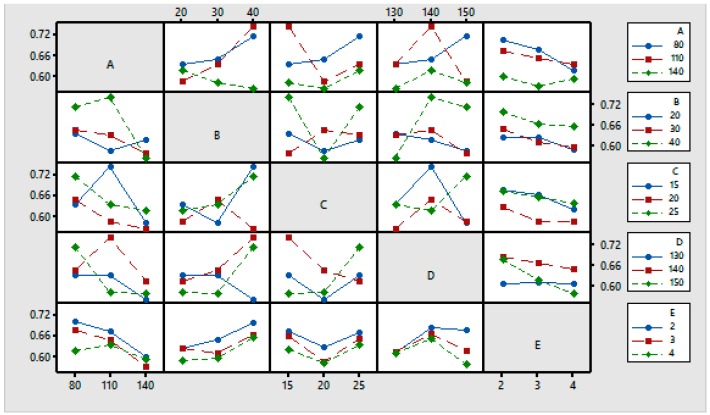
Interaction plots for the means [Response: GRG].

Figure 6 shows the interdependence or interaction among the chosen input parameters for the GRG. The interaction effects of the input parameters can be determined by evaluating the non-parallel and parallel lines in the plot. A good interaction exists between the parameters the lines are non-parallel. On the other hand, a poor interaction exists if the lines are parallel.

An ANOVA was performed for the GRG data. Table 13 shows that the resin concentration has the highest influence (28.27%) on the GRG followed by the polyurethane softener (17.75%), the catalyst concentration (16.32%), the curing temperature (14.27%), and the curing time (7.90%). All the parameters have a significant effect on the process because their p-values are less than 0.05 (95% confidence level).

**Figure 7:**
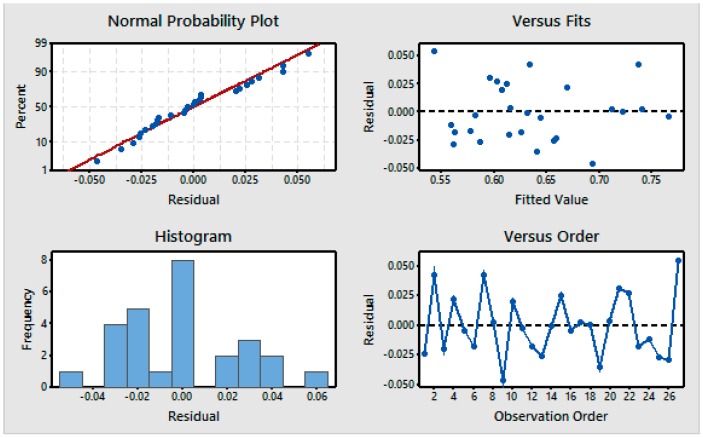
Residual plots for the means [Response: GRG].

The four residual graphs for the GRG are shown in Figure 7. The normal probability plots indicate that the residuals exhibit a nearly linear response. The histogram shows a normal distribution of the weighted GRG for the 27 observations. The plots of the residuals versus the fitted values indicate that residuals are randomly distributed, indicating no sequential association and variance in the error terms. The plots of the residuals versus the order of the data show that the residuals are randomly distributed around zero. Therefore, there is no association with the parameters that would require additional investigation of the error [39].

**Figure 8:**
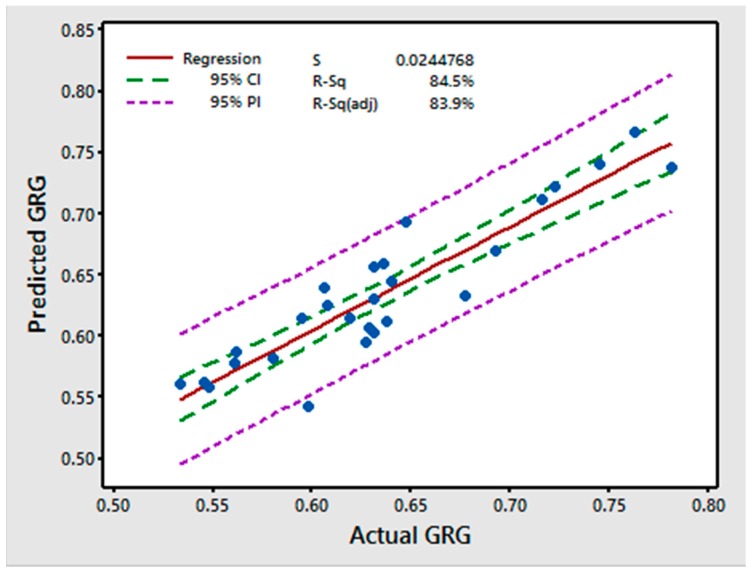
Fitted lines for the actual and predicted GRG.

Figure 8 shows the plot of the predicted versus the actual values. The results indicate that the model is adequate because the residuals are close to the diagonal line. The Pearson correlation coefficient between the predicted GRG values and observed GRG values was 0.8450 with a *p*-value of 0.000, indicating a strong predictive capability of the GRG regression model [40].

### 3.5. Confirmation Test

In the final step of the Taguchi-based GRA, confirmation experiments of the control factors at the optimal and random levels were conducted to verify the accuracy of the optimization and to determine the improvements in the responses [41]. The purpose of the confirmation test is to validate the conclusions drawn following the analysis. Once the optimum levels of the process parameters have been selected, the final step is to predict and verify the improvement in the performance characteristics using the optimum level of the processing parameters. Table 14 shows the results of the confirmation experiments using the optimal processing parameters for the CRA, TE, and WI. The predicted GRG values were calculated using Equation (8). The improvement in the GRG is 0.103676. The confirmation tests exhibit a good agreement between the predicted performance and the actual performance. Additionally, the experimental results confirmed the validity of the applied Taguchi-grey method for improving the performances of the physical properties optimizing the resin finishing parameters.

### 3.6. Structural Characterization

**Figure 9:**
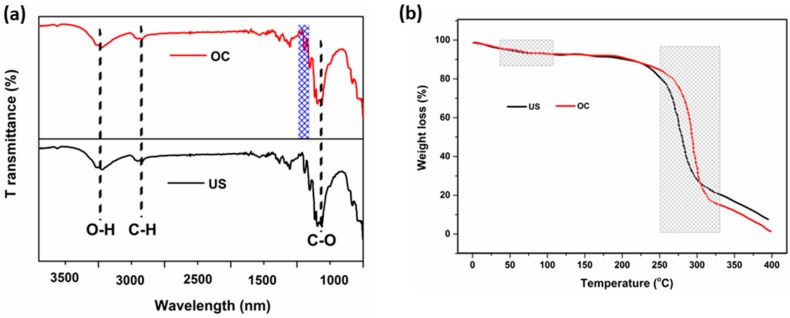
(**a**) FTIR and (**b**) TGA (in nitrogen) spectra of the untreated sample (US) and optimized condition (OC) sample.

The chemical characterization of the treated samples was performed by FTIR spectroscopy, as shown in Figure 9a. FTIR spectrum of the untreated sample (US) shows well-known bands associated with pure cellulose, namely OH stretching at 3364 cm^−1^, CH stretching at 2891 cm^−1^, adsorbed water at 1645 cm^−1^, ring-breathing at 1157 cm^−1^, and C–O stretching at 1026 cm^−1^ [42]. Compared with the untreated sample, the grey optimized condition (OC) of the finished cotton fabric exhibits a new peak at 1201 cm^−1^ to 1322 cm^−1^ (characteristic band of crosslinking agent attached to the fibers, which is shown in Figure 9a blue region) and the absorption at 1648 cm^−1^ (absorption band related to the cellulose fibers) indicates that 31% of the fibers have been grafted during the resin finishing process.

The TGA plots of the US and grey OC cotton fabric are shown in Figure 9b. The minor weight losses observed in the range of 30 °C to 100 °C are attributed to the evaporation of moisture from the samples [43]. The second stage degradation, between 250 °C and 330 °C corresponds to the dissociation of the hydrogen bonding from the cellulose interchain, which is strongly formed among the –OH functional groups. The thermal stability is significantly higher for the treated fabric than for the untreated sample. This is attributed to the interpenetrating polymer network technology that stabilizes the cotton fabric because of the more compact structure resulting from the introduction of the crosslinker. It is known that crosslinked cotton has a higher thermal stability than untreated cotton due to effective crosslinks as indicated by the higher percentage of the residues [44].

**Figure 10:**
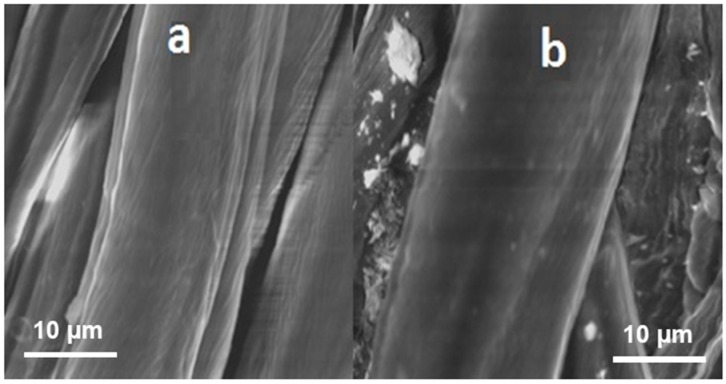
SEM images of (**a**) untreated sample (US) and (**b**) optimized condition (OC) sample.

As can be seen in Figure 10a, the untreated fibers have rough surface with a lot of grooves and imperfections on surface. When comparing Figure 10a to Figure 10b, it could be found that fibers were coated with resin and there were a lot of remarkable cross-linking between adjacent fibers after finishing. It is well known that hydroxyl groups in modified dihydroxyethyleneurea resin react with hydroxyls groups of two cellulose chains as well as those of one chain.

## 4. Conclusions

This study proposes an approach that integrates the Taguchi method and the GRA to identify the optimal combination of processing parameters required to meet multiple quality objectives in resin finishing. The optimum level values of the parameters for resin finishing are the concentrations of the resin (80 g·L^−1^), the polyethylene softener (40 g·L^−1^), and the catalyst (25 g·L^−1^), as well as the curing temperature (140 °C) and the curing time (2 min). The ANOVA results for the GRG indicate a ranking of the input parameters of resin concentration, polyethylene softener, catalyst, curing temperature, and curing time. Therefore, the resin concentration is the most significant parameter for the GRG for resin finishing. All process parameters have a *p*-value of less than 0.05, which means they are all significant. The improvement in the GRG from the initial parameter combination (A_3_B_1_C_3_D_2_E_1_) to the optimal parameter combination (A_1_B_3_C_3_D_2_E_1_) is 0.103676. Moreover, an improvement in the thermal stability was achieved for the optimized fabric.

## Figures and Tables

**Table 1:** Parameters and their levels.

Symbol	Process Parameters	Unit	Level 1	Level 2	Level 3
A	Resin (Knittex RCT)	g·L^−1^	80	110	140
B	Polyethylene Softener	g·L^−1^	20	30	40
C	Catalyst(Knittex^®^ Mo)	g·L^−1^	15	20	25
D	Curing temperature	°C	130	140	150
E	Curing time	min	2	3	4

**Table 2:** Experimental layout using an (L_27_) OA and multi-performance results.

Exp. No.	A	B	C	D	E	CRA (^o^) W + F	S/N Ratio (dB)	TE (gf) W + F	S/N Ratio (dB)	WI	S/N Ratio (dB)
1	1	1	1	1	1	210.2	46.4527	1700.2	64.6100	70.2	36.9267
2	1	1	1	1	2	212.4	46.5431	1700.6	64.6120	70.7	36.9884
3	1	1	1	1	3	217.1	46.7332	1690.2	64.5588	69.3	36.8147
4	1	2	2	2	1	218.2	46.7771	1710.4	64.6620	70.1	36.9144
5	1	2	2	2	2	218.6	46.7930	1700.6	64.6120	69.6	36.8522
6	1	2	2	2	3	220.8	46.8800	1690.6	64.5608	69.1	36.7896
7	1	3	3	3	1	219.4	46.8247	1720.2	64.7116	70.8	37.0007
8	1	3	3	3	2	220.2	46.8563	1710.6	64.6630	70.3	36.9391
9	1	3	3	3	3	222.4	46.9427	1700.8	64.6131	69.2	36.8021
10	2	1	2	3	1	223.2	46.9739	1680.2	64.5072	69.7	36.8647
11	2	1	2	3	2	225.2	47.0514	1670.4	64.4564	68.4	36.7011
12	2	1	2	3	3	226.4	47.0975	1660.6	64.4053	67.1	36.5345
13	2	2	3	1	1	222.6	46.9505	1690.4	64.5598	69.4	36.8272
14	2	2	3	1	2	222.2	46.9349	1680.4	64.5083	69.9	36.8895
15	2	2	3	1	3	227.2	47.1282	1660.2	64.4032	69.8	36.8771
16	2	3	1	2	1	228.4	47.1739	1670.2	64.4554	71.2	37.0496
17	2	3	1	2	2	227.6	47.1434	1680.8	64.5103	70.9	37.0129
18	2	3	1	2	3	232.2	47.3172	1640.2	64.2979	70.4	36.9515
19	3	1	3	2	1	232.4	47.3247	1610.4	64.1387	68.1	36.6629
20	3	1	3	2	2	233.2	47.3546	1630.6	64.2469	67.2	36.5474
21	3	1	3	2	3	234.2	47.3917	1600.1	64.0829	67.5	36.5861
22	3	2	1	3	1	232.6	47.3322	1610.9	64.1414	68.8	36.7518
23	3	2	1	3	2	233.4	47.3620	1590.3	64.0296	65.2	36.2850
24	3	2	1	3	3	232.7	47.3359	1610.7	64.1403	64.3	36.1642
25	3	3	2	1	1	230.2	47.2421	1580.4	63.9753	68.6	36.7265
26	3	3	2	1	2	230.6	47.2572	1560.8	63.8669	67.7	36.6118
27	3	3	2	1	3	232.8	47.3397	1580.6	63.9764	68.2	36.6757

**Table 3:** Response table for S/N ratios (CRA).

Level	A	B	C	D	E
1	46.76	46.99	47.04	46.95	47.01
2	47.09	47.05	47.05	47.13	47.03
3	47.33	47.12	47.08	47.09	47.13
Delta	0.57	0.13	0.03	0.17	0.12
Rank	1	3	5	2	4

**Table 4:** ANOVA for S/N ratio (CRA).

Source	DF	SS	MS	F	*p*	Remarks	P (%)
A	2	1.47805	0.739025	340.36	0.000	significant	81.09
B	2	0.07667	0.038333	17.65	0.000	significant	4.21
C	2	0.00694	0.003468	1.60	0.233	not significant	0.38
D	2	0.15002	0.075012	34.55	0.000	significant	8.23
E	2	0.07624	0.038122	17.56	0.000	significant	4.18
Residual Error	16	0.03474	0.002171				1.91
Total	26	1.82266					

**Table 5:** Response table for the S/N ratios (TE).

Level	A	B	C	D	E
1	64.62	64.40	64.37	64.34	64.42
2	64.46	64.40	64.34	64.40	64.39
3	64.07	64.34	64.44	64.41	64.34
Delta	0.56	0.06	0.10	0.07	0.08
Rank	1	5	2	4	3

**Table 6:** ANOVA for the S/N ratio (TE).

Source	DF	SS	MS	F	*p*	Remarks	P (%)
A	2	1.46601	0.733003	216.10	0.000	significant	89.30
B	2	0.02223	0.011116	3.28	0.064	not significant	1.35
C	2	0.04655	0.023276	6.86	0.007	significant	2.84
D	2	0.02270	0.011348	3.35	0.061	not significant	1.38
E	2	0.02983	0.014914	4.40	0.030	significant	1.82
Residual Error	16	0.05427	0.003392				3.31
Total	26	1.64158					

**Table 7:** Response table for the S/N ratios (WI).

Level	A	B	C	D	E
1	36.89	36.74	36.77	36.82	36.86
2	36.86	36.71	36.74	36.82	36.76
3	36.56	36.86	36.79	36.67	36.69
Delta	0.34	0.16	0.05	0.15	0.17
Rank	1	3	5	4	2

**Table 8:** ANOVA for the S/N ratio (WI).

Source	DF	SS	MS	F	*p*	Remarks	P (%)
A	2	0.61015	0.305076	27.05	0.000	significant	51.42
B	2	0.12580	0.062900	5.58	0.015	significant	10.60
C	2	0.01199	0.005996	0.53	0.598	not significant	1.01
D	2	0.12697	0.063483	5.63	0.014	significant	10.70
E	2	0.13119	0.065596	5.82	0.013	significant	11.06
Residual Error	16	0.18048	0.011280				15.21
Total	26	1.18658					

**Table 9:** Sequence of the performance characteristics after data pre-processing.

Exp. No.	CRA (deg)	TE (gf)	WI
Ref. sequence	1.0000	1.0000	1.0000
1	0.0000	0.8750	0.8550
2	0.0920	0.8770	0.9280
3	0.2880	0.8120	0.7250
4	0.3330	0.9390	0.8410
5	0.3500	0.8770	0.7680
6	0.4420	0.8140	0.6960
7	0.3830	1.0000	0.9420
8	0.4170	0.9400	0.8700
9	0.5080	0.8780	0.7100
10	0.5420	0.7490	0.7830
11	0.6250	0.6880	0.5940
12	0.6750	0.6260	0.4060
13	0.5170	0.8130	0.7390
14	0.5000	0.7500	0.8120
15	0.7080	0.6240	0.7970
16	0.7580	0.6860	1.0000
17	0.7250	0.7530	0.9570
18	0.9170	0.4980	0.8840
19	0.9250	0.3110	0.5510
20	0.9580	0.4380	0.4200
21	1.0000	0.2470	0.4640
22	0.9330	0.3140	0.6520
23	0.9670	0.1850	0.1300
24	0.9380	0.3130	0.0000
25	0.8330	0.1230	0.6230
26	0.8500	0.0000	0.4930
27	0.9420	0.1240	0.5650

**Table 10:** The deviation sequences.

Deviation Sequences	Δ_0*i*_ (1)	Δ_0*i*_ (2)	Δ_0*i*_ (3)
1	1.0000	0.1255	0.1449
2	0.9083	0.1230	0.0725
3	0.7125	0.1882	0.2754
4	0.6667	0.0615	0.1594
5	0.6500	0.1230	0.2319
6	0.5583	0.1857	0.3043
7	0.6167	0.0000	0.0580
8	0.5833	0.0602	0.1304
9	0.4917	0.1217	0.2899
10	0.4583	0.2509	0.2174
11	0.3750	0.3124	0.4058
12	0.3250	0.3739	0.5942
13	0.4833	0.1870	0.2609
14	0.5000	0.2497	0.1884
15	0.2917	0.3764	0.2029
16	0.2417	0.3137	0.0000
17	0.2750	0.2472	0.0435
18	0.0833	0.5019	0.1159
19	0.0750	0.6888	0.4493
20	0.0417	0.5621	0.5797
21	0.0000	0.7535	0.5362
22	0.0667	0.6857	0.3478
23	0.0333	0.8149	0.8696
24	0.0625	0.6870	1.0000
25	0.1667	0.8770	0.3768
26	0.1500	1.0000	0.5072
27	0.0583	0.8758	0.4348

**Table 11:** The calculated grey relational coefficients and GRGs.

Exp. No.	Grey Relational Coefficient			Grey Relational Grade	S/N Ratio	Order
	WRA (°)	TE (gf)	WI			
1	0.3333	0.7994	0.7753	0.6360	−3.9308	11
2	0.3550	0.8026	0.8734	0.6770	−3.3880	7
3	0.4124	0.7265	0.6449	0.5946	−4.5157	21
4	0.4286	0.8905	0.7582	0.6924	−3.1924	6
5	0.4348	0.8026	0.6832	0.6402	−3.8738	9
6	0.4724	0.7292	0.6216	0.6077	−4.3255	18
7	0.4478	1.0000	0.8961	0.7813	−2.1438	1
8	0.4615	0.8925	0.7931	0.7157	−2.9052	5
9	0.5042	0.8042	0.6330	0.6472	−3.7798	8
10	0.5217	0.6658	0.6970	0.6282	−4.0383	15
11	0.5714	0.6154	0.5520	0.5796	−4.7371	22
12	0.6061	0.5721	0.4570	0.5451	−5.2712	26
13	0.5085	0.7279	0.6571	0.6312	−3.9973	13
14	0.5000	0.6669	0.7263	0.6311	−3.9982	14
15	0.6316	0.5705	0.7113	0.6378	−3.9062	10
16	0.6742	0.6145	1.0000	0.7629	−2.3508	2
17	0.6452	0.6692	0.9200	0.7448	−2.5594	3
18	0.8571	0.4991	0.8118	0.7227	−2.8214	4
19	0.8696	0.4206	0.5267	0.6056	−4.3560	19
20	0.9231	0.4708	0.4631	0.6190	−4.1665	17
21	1.0000	0.3989	0.4825	0.6271	−4.0527	16
22	0.8824	0.4217	0.5897	0.6313	−3.9958	12
23	0.9375	0.3802	0.3651	0.5609	−5.0216	24
24	0.8889	0.4212	0.3333	0.5478	−5.2272	25
25	0.7500	0.3631	0.5702	0.5611	−5.0190	23
26	0.7692	0.3333	0.4964	0.5330	−5.4656	27
27	0.8955	0.3634	0.5349	0.5979	−4.4668	20

**Table 12:** Response table for the GRG.

Level	A	B	C	D	E
1	0.6658	0.6125	0.6531	0.6111	0.6589
2	0.6537	0.6201	0.5984	0.6692	0.6335
3	0.5871	0.6741	0.6551	0.6263	0.6142
Delta	0.0787	0.0616	0.0567	0.0581	0.0447
Rank	1	2	4	3	5

**Table 13:** ANOVA for the GRG.

Source	DF	SS	MS	F	*p*	Remarks	P (%)
A	2	0.032330	0.016165	14.59	0.000	significant	28.27
B	2	0.020304	0.010152	9.16	0.002	significant	17.75
C	2	0.018660	0.009330	8.42	0.003	significant	16.32
D	2	0.016319	0.008160	7.37	0.005	significant	14.27
E	2	0.009036	0.004518	4.08	0.037	significant	7.90
Residual Error	16	0.017725	0.001108				15.50
Total	26	0.114374					

**Table 14:** Results of the confirmation experiment.

Response	Initial Parameters	Prediction	Confirmation Experiment
Level	A_3_B_1_C_3_D_2_E_1_	A_1_B_3_C_3_D_2_E_1_	A_1_B_3_C_3_D_2_E_1_
CRA	232.4		231.6
TE	1610.4		1674.6
WI	68.1		69.5
Grey relational grade	0.605621	0.780997	0.709298
Improvement in grey relational grade	0.103676		

## Abbreviations

AATCC	American Association of Textile Chemists and Colorists
ANOVA	Analysis of variance
CRA	Crease recovery angle
DF	Degree of freedom
F	F-test value
FTIR	Fourier-transform infrared
GRA	Grey relational analysis
GRG	Grey relational grade
MS	Mean square
*p*	*p*-value: probability value
P (%)	Percentage contribution
R-Sq (adj)	adjusted R-squared statistic: correlation
R-Sq (R^2^)	R-squared statistic
SEM	Scanning electron microscope
SS	Sum of square
S/N ratio	Signal-to-noise ratio
TE	Tearing strength
TGA	Thermogravimetric analysis
WI	Whiteness index

## References

[1] Goynes W.R., Rollins M.L. (1971). A scanning electron-microscope study of washer-dryer abrasion in cotton fibers. Text. Res. J..

[2] Cai Y., Pailthorpe M.T., David S.K. (1999). A new method for improving the dyeability of cotton with reactive dyes. Text. Res. J..

[3] Teli M., Sheikh J., Bhavsar P. (2013). Multifunctional finishing of cotton using chitosan extracted from bio-waste. Int. J. Biol. Macromol..

[4] Shi F., Wang Y. (2009). Modelling crease recovery behaviour of woven fabrics. J. Text. Inst..

[5] Fan J. (2001). The interrelationship between fabric crease recovery and pressing performance. Int. J. Cloth. Sci. Technol..

[6] Harifi T., Montazer M. (2012). Past, present and future prospects of cotton cross-linking: New insight into nano particles. Carbohydr. Polym..

[7] Oakes J., Gratton P. (1998). Kinetic investigations of azo dye oxidation in aqueous media. J. Chem. Soc. Perkin Trans..

[8] Nalbant M., Gökkaya H., Sur G. (2007). Application of Taguchi method in the optimization of cutting parameters for surface roughness in turning. Mater. Des..

[9] Chakravorty R., Gauri S.K., Chakraborty S. (2012). Optimization of correlated responses of EDM process. Mater. Manuf. Process..

[10] Yuvaraj N., Pradeep Kumar M. (2015). Multiresponse optimization of abrasive water jet cutting process parameters using TOPSIS approach. Mater. Manuf. Process..

[11] Kuo Y., Yang T., Huang G.-W. (2008). The use of grey relational analysis in solving multiple attribute decision-making problems. Comput. Ind. Eng..

[12] Julong D. (1989). Introduction to grey system theory. J. Grey Syst..

[13] Ng D.K.W. (1994). Grey system and grey relational model. ACM SIGICE Bull..

[14] Siddiquee A.N., Khan Z.A., Mallick Z. (2010). Grey relational analysis coupled with principal component analysis for optimisation design of the process parameters in in-feed centreless cylindrical grinding. Int. J. Adv. Manuf. Technol..

[15] Hasani H., Tabatabaei S.A., Amiri G. (2012). Grey relational analysis to determine the optimum process parameters for open-end spinning yarns. J. Eng. Fibers Fabr..

[16] Raza Z.A., Ahmad N., Kamal S. (2014). Multi-response optimization of rhamnolipid production using grey rational analysis in Taguchi method. Biotechnol. Rep..

[17] Rehman A., Raza Z.A., Masood R., Hussain M.T., Ahmad N. (2015). Multi-response optimization in enzymatic desizing of cotton fabric under various chemo-physical conditions using a Taguchi approach. Cellulose.

[18] Hussain T., Arain F.A., Malik Z.A. (2017). Use of Taguchi Method and Grey Relational Analysis to Optimize Multiple Yarn Characteristics in Open-End Rotor Spinning. Autex Res. J..

[19] Deepanraj B., Sivasubramanian V., Jayaraj S. (2017). Multi-response optimization of process parameters in biogas production from food waste using Taguchi–Grey relational analysis. Energy Convers. Manag..

[20] Vasantharaj K., Jerold M., Deepanraj B., Velan M., Sivasubramanian V. (2017). Assessment of a sulfidogenic system utilizing microalgal biomass of Chlorella pyrenoidosa as an electron donor: Taguchi based grey relational analysis. Int. J. Hydrogen Energy.

[21] Ahmad N., Kamal S., Raza Z.A., Hussain T., Anwar F. (2016). Multi-response optimization in the development of oleo-hydrophobic cotton fabric using Taguchi based grey relational analysis. Appl. Surf. Sci..

[22] (1974). Wrinkle Recovery of Fabrics.

[23] ASTM D1424 (1978). Tearing Strength of Fabrics.

[24] ASTM E313 (2004). Standard Practice for Calculating Yellowness and Whiteness Indices from Instrumentally Measured Color Coordinates.

[25] Tsui K.-L. (1992). An overview of Taguchi method and newly developed statistical methods for robust design. IIE Trans..

[26] Mavruz S., Oğulata R.T. (2010). Taguchi approach for the optimisation of the bursting strength of knitted fabrics. FIbres Text. East. Eur..

[27] Chang C.-K., Lu H.S. (2007). Design optimization of cutting parameters for side milling operations with multiple performance characteristics. Int. J. Adv. Manuf. Technol..

[28] Khanna R., Kumar A., Garg M.P., Singh A., Sharma N. (2015). Multiple performance characteristics optimization for Al 7075 on electric discharge drilling by Taguchi grey relational theory. J. Ind. Eng. Int..

[29] Yang Y.-K. (2006). Optimization of injection-molding process for mechanical and tribological properties of short glass fiber and polytetrafluoroethylene reinforced polycarbonate composites with grey relational analysis: A case study. Polym. Plast. Technol..

[30] Nguyen T.C., Miska S., Saasen A., Maxey J. (2014). Using Taguchi and ANOVA methods to study the combined effects of drilling parameters on dynamic barite sag. J. Petrol. Sci. Eng..

[31] Senthilkumar N., Tamizharasan T., Anandakrishnan V. (2014). Experimental investigation and performance analysis of cemented carbide inserts of different geometries using Taguchi based grey relational analysis. Measurement.

[32] Hassan Y.M.E., EL-Salmawy A., Almetwally A. (2010). Performance of woven fabrics containing spandex. Indian Text. J..

[33] Dhamija S., Chopra M. (2007). Tearing strength of cotton fabrics in relation to certain process and loom parameters. Indian J. Fibre Text. Res..

[34] Montazer M., Afjeh M.G. (2007). Simultaneous x-linking and antimicrobial finishing of cotton fabric. J. Appl. Polym. Sci..

[35] Hung O.N., Chan C.K., Kan C.W., Yuen C.W.M. (2017). An analysis of some physical and chemical properties of CO2 laser-treated cotton-based fabrics. Cellulose.

[36] Fouda M.M.G., El Shafei A., Sharaf S., Hebeish A. (2009). Microwave curing for producing cotton fabrics with easy care and antibacterial properties. Carbohydr. Polym..

[37] Baskaran V., Nachiappan S., Rahman S. (2012). Indian textile suppliers' sustainability evaluation using the grey approach. Int. J. Prod. Econ..

[38] Kuo C.-F.J., Tu H.-M. (2009). Gray relational analysis approach for the optimization of process setting in textile calendering. Text. Res. J..

[39] Sudhakara D., Prasanthi G. (2017). Parametric Optimization of Wire Electrical Discharge Machining of Powder Metallurgical Cold Worked Tool Steel using Taguchi Method. J. Inst. Eng. India Ser. C.

[40] Hussain T., Ali S., Qaiser F. (2010). Predicting the crease recovery performance and tear strength of cotton fabric treated with modified *N*-methylol dihydroxyethylene urea and polyethylene softener. Color. Technol..

[41] Sarıkaya M., Güllü A. (2015). Multi-response optimization of minimum quantity lubrication parameters using Taguchi-based grey relational analysis in turning of difficult-to-cut alloy Haynes 25. J. Clean. Prod..

[42] Garside P., Wyeth P. (2003). Identification of cellulosic fibres by FTIR spectroscopy-thread and single fibre analysis by attenuated total reflectance. Stud. Conserv..

[43] Wang M., She Y., Xiao Z., Hu J., Zhou R., Zhang J. (2014). The green adsorption of chitosan tripolyphosphate nanoparticles on cotton fiber surfaces. Carbohydr. Polym..

[44] Trask-Morrell B.J., Kottes Andrews B.A. (1994). Thermoanalytical Study of Durable Press Reactant Levels on Cotton Fabrics: Part I: Nonformaldehyde Polycarboxylic Acids. Text. Res. J..

